# Genome Sequence of *Arthrobacter* Phage Sourignavong

**DOI:** 10.17912/micropub.biology.001462

**Published:** 2025-03-14

**Authors:** Ian T. Rife, Nana Sannomiya, Katie N. Thai, Cori B. Williams, Raelynn A. Amasol-Tanoura, Jennifer H. Arca, Gillian A. Bradley, Avery C. Catlin, Saige A. Edwards, Sophia F. Faria, Maximilliano Fuentes-Ayala, Lola M. Gantenbein, Alexis M. Haiges, Hailey E. Hart, Ayden K. Herrera, Paisley J. Karlin, Landon H. Schumaker, Emily J. Velasquez, Hannah E. Moon, Megan L. Porter

**Affiliations:** 1 School of Life Sciences, University of Hawaiʻi at Mānoa, Honolulu, Hawaii, United States

## Abstract

We report the genome sequence of phage Sourignavong isolated from soil in Oklahoma City, Oklahoma using *Arthrobacter* sp. ATCC 21022. The 15,625 bp genome contains 27 predicted protein coding genes, including two predicted endolysin enzyme genes. Sourignavong is assigned to actinobacteriophage cluster AN.

**Figure 1. Sourignavong phage morphology:**
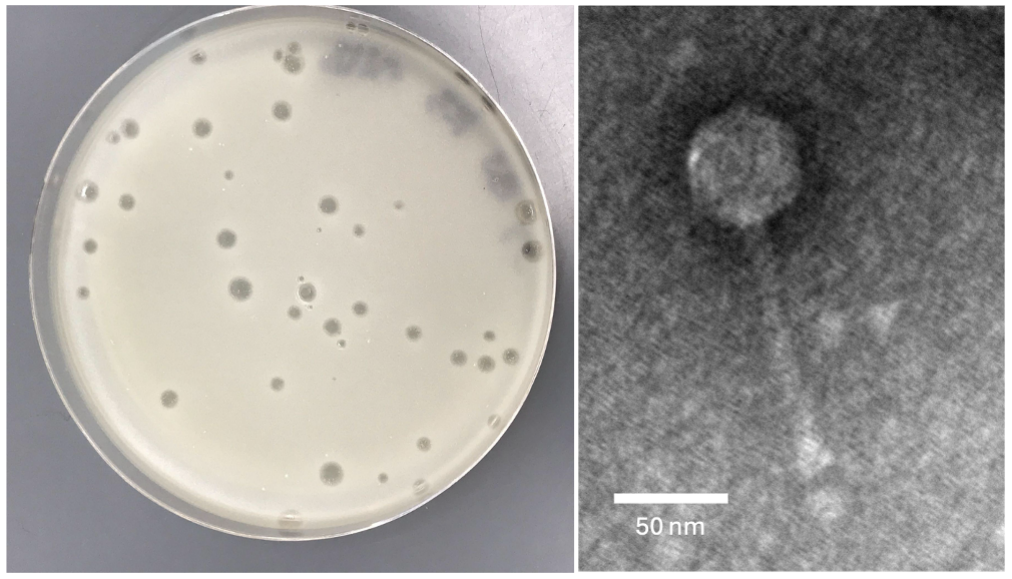
Plaque morphology of Sourignavong on *Arthrobacter* sp. ATCC 21022 host (left). Plaque sizes range in size from 0.8-4.0 mm. Transmission electron microscopy image of Sourignavong (right). Scale bar is 50 nm.

## Description

Uncovering the diversity of phages is an important endeavor in the development of phage as therapeutics for bacterial infections, particularly those that are resistant to antibiotics (Hatfull 2022). Here, we present phage Sourignavong that infects *Arthrobacter* sp. ATCC 21022.

Sourignavong was discovered in Oklahoma City, OK from moist soil surrounding a decaying tree stump (GPS: 35.515833 N, 97.530556 W), using standard procedures (Zorawik et al., 2024). Briefly, the soil sample was suspended in PYCa medium (peptone, yeast extract, calcium), then spun and the supernatant filtered (0.22 um pore size), before the filtrate was inoculated with *Arthrobacter* sp. and incubated with shaking at 30˚C for 72 hours. The culture was then refiltered and the filtrate plated on top agar with *Arthrobacter* sp. After incubation at 30˚C, Sourignavong formed clear plaques 0.8 – 4.0 millimeter in diameter (n=38; Fig.1) and was subsequently purified through three rounds of plating. Negative stain (1% uranyl acetate) transmission electron microscopy revealed a siphovirus morphology with a capsid size of 55 nm and a tail length of 135 nm (n=1; Fig. 1).

DNA was extracted from a liquid lysate using a Promega Wizard DNA Clean-up kit and prepared for sequencing using the NEB Ultra II Library Kit (Zorawik et al., 2024). The Sourignavong genome was sequenced by Illumina sequencing technology (MiSeq, v3 reagents), resulting in 27,191 150-base reads. Raw reads were assembled in Newbler v2 (Roche), resulting in a single genomic contig with 224-fold coverage. The genome was circular, with a total genome length of 15,625 bps, and a GC content of 60.30%.

The auto annotation of the Sourignavong genome was completed using Glimmer v3.02b (Delcher et al., 2007) and GeneMark v3.25 (Besemer and Borodovsky, 2005). Start sites were manually verified using the Phage Evidence Collection and Annotation Network (PECAAN) v.20240320 (https://discover.kbrinsgd.org), Starterator v562 (http://phages.wustl.edu/starterator), and Phamerator v560 (Cresawn et al., 2011). Potential functions for predicted protein coding genes were designated based on top hits from HHpred (PDB_mmCIF70, SCOPe70, Pfam-A, and NCBI_Conserved_Domains databases) (Zimmermann et al., 2018) as well as BLAST searches against two databases (NCBI, Actinobacteriophage Proteins) (Altschul et al., 1990; Camacho et al., 2009; Russell and Hatfull, 2017). ARAGORN v1.2.38 (Laslett and Canback, 2004) was used to identify potential tRNAs. Default parameters were used for all software.

Through this annotation process, Sourignavong has 27 predicted genes, of which 15 were assigned putative functions, with no tRNAs. Based on gene content similarity (GCS) to phages in the Actinobacteriophage database (Russell and Hatfull, 2017), Sourignavong is assigned to phage cluster AN (Pope et al. 2017; Russel and Hatfull, 2017). As with previously characterized cluster AN phages, the majority of Sourignavong’s small genome encodes structure, assembly, and lysis genes, including a fused protease-capsid gene and two consecutive endolysin genes (Klyczek et al. 2017). No immunity repressor or integrase functions could be identified, suggesting Sourignavong is unlikely to establish lysogeny.

**Nucleotide sequence accession numbers**

Sourignavong is available at GenBank with Accession No. PP978872 and Sequence Read Archive (SRA) SRX24892107 .
